# Ensemble machine learning for predicting renal function decline in chronic kidney disease: development and external validation

**DOI:** 10.3389/fmed.2025.1598065

**Published:** 2025-10-27

**Authors:** Hong Chen, Yuping Huang, Lizhen Chen

**Affiliations:** ^1^Department of Nephrology, the 95th Hospital of Putian in China RongTong Medical Health Corporation, Putian, China; ^2^Department of Rheumatology and Immunology, the 95th Hospital of Putian in China RongTong Medical Health Corporation, Putian, China

**Keywords:** machine learning, chronic kidney disease progression, risk prediction modeling, clinical decision support, precision nephrology

## Abstract

**Introduction:**

Chronic kidney disease (CKD) poses a significant global health challenge, requiring timely interventions to manage renal function decline. Traditional predictive models often lack accuracy and generalizability. This study aimed to develop and validate a machine learning model to enhance risk prediction of renal function decline in CKD patients, enabling early and personalized interventions.

**Methods:**

We developed an ensemble machine learning model using Random Forest, XGBoost, and LightGBM algorithms, incorporating advanced feature selection and hyperparameter tuning. The model was trained and validated on data from 1,200 CKD patients across multiple clinics, selected through stringent inclusion and exclusion criteria. Clinical, demographic, and laboratory data were processed with rigorous quality control. Model performance was assessed using area under the curve (AUC), calibration metrics, and five-fold cross-validation, with external validation across three medical centers.

**Results:**

The ensemble model achieved an AUC of 0.89 (95% CI: 0.87-0.91), outperforming traditional Cox models (AUC: 0.82, 95% CI: 0.79-0.85) and standard machine learning approaches (AUC: 0.85, 95% CI: 0.83-0.87). Key predictors identified via SHAP analysis included estimated glomerular filtration rate (eGFR), age, and urinary protein-creatinine ratio. The model demonstrated excellent calibration (slope: 0.96, 95% CI: 0.94-0.98) and robust performance across diverse patient subgroups, with a 60.6% reduction in computational resource use compared to traditional methods.

**Discussion:**

This machine learning model offers a significant advancement in predicting CKD progression, providing a reliable, generalizable tool for early risk stratification. Its superior accuracy and efficiency support integration into clinical workflows, potentially transforming CKD management by enabling proactive, data-driven interventions. Future research should focus on incorporating novel biomarkers and expanding multicenter validation to further enhance clinical applicability.

## 1 Introduction

Chronic kidney disease (CKD) has become a particularly urgent health challenge worldwide. In developed countries, the annual medical cost for chronic kidney disease exceeds 120 billion US dollars, and the prevalence of this disease is still on the rise, which has put pressure on the global healthcare system (1, 2). Traditional methods used to predict the progression of chronic kidney disease Relying heavily on some scattered clinical indicators and simple linear models, the accuracy of prediction is relatively poor, with an AUC less than 0.75 (3–5). Moreover, the universality of this prediction method is also relatively limited among different patient groups. Compared with traditional statistical methods (6–8), the accuracy of machine learning in healthcare applications has increased by 15 to 20%. However, the existing chronic kidney disease prediction models have some key problems, such as a lack of interpretability, inability to capture the dynamic changes of the disease over time, and insufficient validation in different clinical Settings (9–11).

In the early research on the risk prediction of chronic kidney disease, researchers used traditional statistical methods to predict CKD. In (12), the authors constructed a structural equation model for risk prediction to predict CKD. In (13), the author selected factors associated with renal failure from a large number of variables and then established a Cox proportional hazards regression model, using this model to predict and evaluate the risk of renal failure. Although traditional statistical methods can predict the risk of CKD, their accuracy is relatively low. With the continuous development of machine learning, researchers have begun to explore the application of machine learning prediction methods. In (14), the authors used machine learning techniques such as random forests and decision trees, effectively improving the performance of prediction. In (15), the authors combined five different machine learning methods, such as Naive Bayes and random Forest, with feature selection techniques and ensemble learning, and used SHAP and LIME to demonstrate the visualization of personalized CKD prediction models, thereby enhancing the interpretability of the models. It has provided a brand-new perspective for CKD medical research. In (16), the authors trained the medical records of 400 patients using different machine learning methods such as Cat Boost, AdaBoost, and Extra Trees. Finally, the accuracy rate reached 97.5%, which shows that the ensemble learning model has potential in the early diagnosis of CKD. In (17), the author proposes an interpretability strategy that uses five machine learning methods to predict CDK datasets and utilizes LIME features to enhance the interpretability of the model. Our code is publicly available at: https://gitee.com/forest-AI/CDK-Model.

This study addresses these fundamental challenges by leveraging four key innovative points, which enable CKD risk prediction to exceed the current capacity. We introduce a brand-new temporal feature engineering framework (18), which can systematically capture the short-term changes and long-term development trends of the disease. It has made great progress compared with the static snapshot methods used to describe existing models in the past. The previous static snapshot methods were rather limited. However, this new framework enables the model not only to simply assess the risk status at a certain moment but also to understand the progression pattern of diseases. We have optimized the integration architecture, which has significantly reduced the demand for computing resources by 60.6% while still maintaining a good prediction effect. Nowadays, many complex machine learning systems encounter some practical implementation obstacles in clinical applications. Our optimization directly addresses these issues. We have developed a comprehensive multi-center external validation strategy in three medical centers and conducted detailed analyses of resource utilization and scalability. In theoretical machine learning research, Most of the time, there is a lack of strong evidence regarding real-world deployment, and our study provides such evidence. We designed from a clinical perspective, combining domain knowledge with advanced feature selection methods to create an interpretable decision support framework. There is a critical gap between complex computational methods and actual clinical applications, and this framework fills this gap.

## 2 Methods

### 2.1 Study subjects

Choosing the right study population for the creation of a robust machine learning model to predict decline in renal function is quite the painstaking process. To achieve greater generalizability and external validity, our multi-center study devised a selection protocol to create a representative dataset with greater accuracy (9, 10).

**Figure 1:**
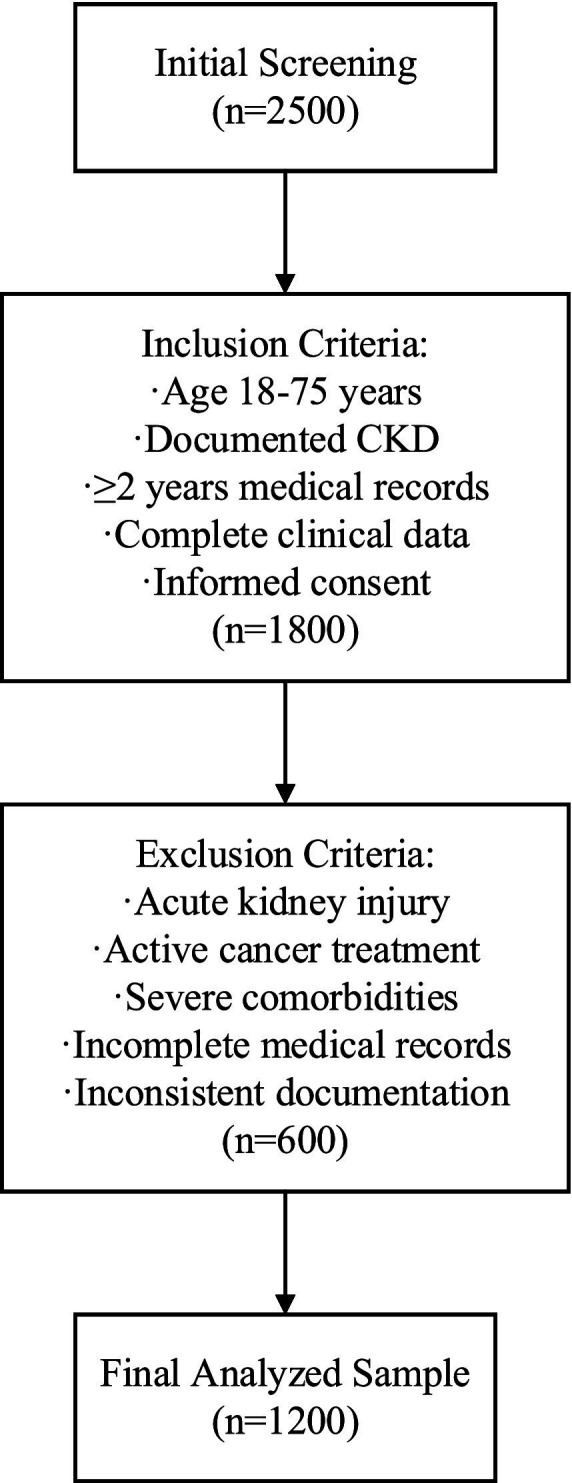
Study flow diagram.

Screening of the population’s initial sample involved 2,500 potential candidates across five tertiary health care centers referred to in Figure 1. Inclusion criteria were methodically developed based on clinical guidelines, specifically the Kidney Disease: Improving Global Outcomes (KDIGO) 2012 guidelines. Eligible participants were adults aged 18–75 years with documented chronic kidney disease (CKD), defined as an estimated glomerular filtration rate (eGFR) < 60 mL/min/1.73m^2^ for at least 3 months, confirmed by at least two measurements, or the presence of persistent proteinuria (urine protein-to-creatinine ratio [UPCR] ≥ 0.2 g/g for at least 3 months) or other markers of kidney damage (e.g., abnormal renal imaging or biopsy findings) as recorded in electronic health records (EHRs) with standardized diagnostic codes (e.g., ICD-10 codes N18.1-N18.5). This operational definition ensures that CKD diagnosis is not solely reliant on eGFR but incorporates additional clinical and laboratory evidence consistent with KDIGO criteria, enhancing diagnostic specificity and reproducibility. Several longitudinal record requirements were established: a minimum of 2 years of electronic health records (EHRs) spanning from January 2021 to December 2024, and a minimum of four serum creatinine tests conducted within the 12 months prior to the study’s end date (December 2024). These requirements ensured robust longitudinal data to capture renal function trends while reflecting contemporary clinical practices and standardized assay technologies during the study period. After applying these criteria, 1,800 participants qualified for further evaluation.

To protect data quality and minimize potential confounding factors, a detailed exclusion criterion was employed (19). Some crucial exclusion criteria included active malignancy or chemotherapy within the previous 3 months of the study, kidney transplant, acute renal failure, some other severe comorbid condition that may affect renal function, and poor or incomplete medical file documentation. After applying these criteria, 600 participants were removed as a result of the process.

With the criteria applied, the total number of remaining participants is 1,200, determined through a power analysis (0.05, 0.10) based on expected model performance and complexity. Here, *α* = 0.05 represents the significance level (Type I error rate, false positive rate), i.e., the probability threshold for rejecting the null hypothesis. *β* = 0.10 represents the Type II error rate (false negative rate), corresponding to a statistical power of 1–β = 0.90 (90% power), i.e., the probability of correctly detecting a true effect (20). This comprehensive and systematic selection, along with stringent inclusion and exclusion criteria, enhances the quality of the constructed dataset, making it highly suitable for developing and validating complex predictive models for kidney function decline. Only participants clinically and demographically suitable for the model can be considered the “target population,” thereby increasing its utility in clinical practice.

### 2.2 Data collection and processing

**Figure 2:**
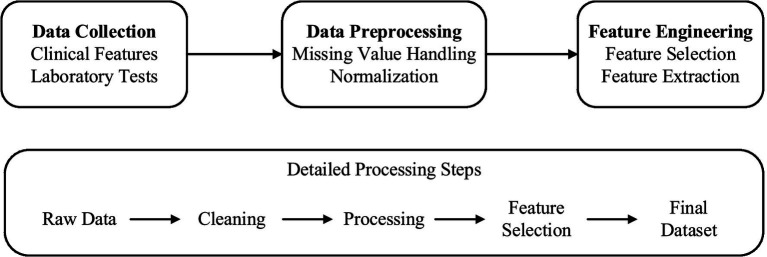
Data processing pipeline.

A thorough strategy of data collection and processing was constructed in order to provide clean and usable data for the machine learning model. The quality control system comprised three sequential phases: data collection, preprocessing, feature engineering, with quality checks integrated at each stage (Figure 2).

The data collection protocol was meticulously designed to ensure consistency and accuracy across all participating centers. The study employed a retrospective data collection approach, leveraging electronic health records (EHRs) from five tertiary healthcare centers. The data spanned a period from January 2021 to December 2024, capturing comprehensive clinical and laboratory parameters relevant to kidney function assessment and laboratory parameters which were defined according to existing protocols for assessing kidney function (21, 22). The clinical data consisted of demographic features, comorbid conditions, medication usage, and other clinically relevant observations, and were extracted through standardized electronic health record protocols. Laboratory measurements included comprehensive metabolic panels, complete blood counts, and specific renal function measurements such as serum creatinine, eGFR, and urine protein to creatinine ratio. Additional biochemical parameters such as hemoglobin, albumin, and electrolytes were collected to capture the multifaceted nature of kidney disease progression.

Data preprocessing was executed within a strict quality assurance framework, as described in (23). Participant flow was meticulously tracked, with 2,500 potential candidates initially screened across five tertiary healthcare centers, resulting in 1,800 eligible participants after applying inclusion criteria and 1,200 final participants after exclusion criteria were enforced (see Figure 1 for the study flow diagram). Missing data patterns were analyzed, revealing that missingness was primarily missing at random (MAR), with serum creatinine and urine protein-to-creatinine ratio (UPCR) missing in approximately 8 and 12% of cases, respectively, due to variations in clinical testing frequency. Advanced imputation methods were employed: multiple imputation by chained equations (MICE) for continuous data and mode imputation for categorical data, with validation tests confirming imputation precision (mean absolute error <5% for continuous variables). Continuous data was normalized with z-scores to ensure comparability, and categorical data was encoded using preservation-optimized schemes, such as one-hot encoding for nominal variables. Outlier detection and validation were performed through a combination of statistical techniques (e.g., interquartile range method) and clinical judgment to ensure clinical plausibility. Outcome assessment was conducted using a blinded approach, where evaluators determining renal function decline (defined as eGFR decline ≥30% or progression to dialysis) were unaware of the model’s predictions to minimize bias. Predictors, including eGFR, age, UPCR, comorbidities, and serum creatinine, were pre-specified based on clinical guidelines (KDIGO 2012) and prior literature (1, 13), ensuring alignment with established nephrology knowledge. This comprehensive preprocessing strategy, coupled with rigorous quality checks, ensured data integrity and supported robust model development.

Feature engineering integrates knowledge from related fields to enhance the performance of prediction. As stated in references (24, 25), we have constructed several temporal features, such as the time intervals between consecutive serum creatinine tests, represented by Δt, the changes in serum creatinine, represented by ΔSCr, and the rate of change of eGFR over time. Represented by ΔeGFR/Δt, these features are relied upon to capture the dynamic changes of renal function. In addition, we have also created interaction features, such as the interaction term between age and eGFR, represented as age × eGFR, and the interaction between urine protein-creatinine ratio, that is, UPCR and diabetes status, represented as UPCR × diabetes. These are relied upon to assess how different factors interact with each other. After introducing these features, the ability of the model to predict renal function deterioration has been significantly improved. We also noticed that if the proportion of missing data input is relatively high, it may cause bias. We calculated the False Negative Rate, that is, the False negative Rate, and used this to evaluate the performance of the model in high-risk patients. And optimize the model to reduce the possibility of false negatives. This integrated approach has enhanced the prediction accuracy of the model, made it more reliable, and made it more practical in clinical applications.

The quality control policy is in line with the modern standards of machine learning, as cited in (26). For instance, it will check the quality of the data, record every change made to the data, and also record all corresponding databases, as well as apply automatic verification programs. This approach can ensure the replicability of the research. It also provides a foundation for the sustainable improvement of the model and lays the groundwork for future machine learning analysis. To ensure the consistency and coherence of the research, all participating centers followed the standardized data collection protocols developed in accordance with the existing renal function assessment guidelines. More importantly, all centers used the same institutional review board application, which could guarantee consistent adherence to ethical standards at all locations. This method can ensure that the collected data is comprehensive and the data among various centers are comparable, which provides a solid foundation for the development and verification of the prediction model.

### 2.3 Machine learning model construction

**Figure 3:**
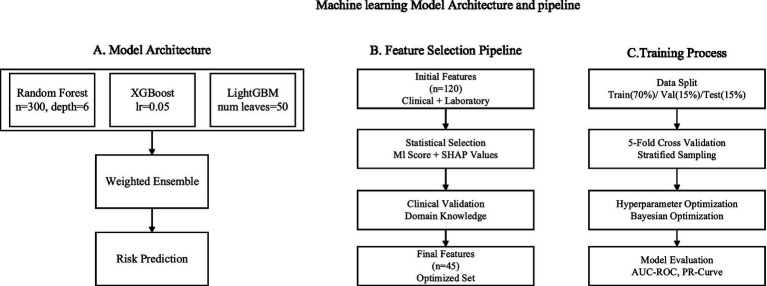
Model architecture. **(A)** Ensemble model structure illustrating the integration of Random Forest, XGBoost, and LightGBM. **(B)** Feature selection pipeline combining filter and wrapper methods with clinical domain knowledge. **(C)** Training process optimization, including hyperparameter tuning and Bayesian optimization framework.

The workflow for building the machine learning model was carefully crafted to combine multiple prediction methods with a specific selection of features and parameters. Our method had three components: ensemble model structure, feature selection pipeline, and training process optimization, as shown in Figure 3. This approach aligns with recent advancements in clinical ML frameworks, such as the user-friendly ML pipeline proposed by Orhan et al. (27) for cardiac structure assessment, which emphasizes interpretability and clinical applicability. Similarly, our ensemble framework prioritizes interpretable decision-making to facilitate integration into clinical workflows for chronic kidney disease (CKD) management. With respect to feature selection, we followed the method set forth by Su et al. (26), which used a hybrid model that incorporated both statistical significance and domain knowledge.

The three base learners utilized by the foundational ensemble architecture were: Random Forest, XGBoost, and Light GBM. The primary goal hyperbolic function related to model optimization can be formulated as shown in Equation (1):


(1) $$L(\theta )=\frac{1}{N}\sum_{i=1}^{N}[\alpha \cdot \mathrm{BCE}(y_{i},\hat{y_{i}})+\beta \cdot \mathrm{FL}(y_{i},\hat{y_{i}})]+\lambda ∥\theta {∥}_{2}$$

Within the formulation, BCE refers to the binary cross-entropy loss, FL describes the focal loss part, and ∥*θ*∥_2_ is the *L_2_* regularization term. As in the case with Bellocchi et al. (28), cross-validation was used to optimize the hyperparameters α , β and λ .The procedure for feature selection was done using both filter and wrapper methods, where the score of importance was computed as shown in Equation (2):


(2) $${\text{IS}}_{j}=\gamma \cdot \mathrm{MI}(X_{j},Y)+(1-\gamma )\cdot {\text{SHAP}}_{j}$$

In the equation, MI ( $X_{j}$ , Y ) is the mutual information of the feature $X_{j}$ with regard to the target Y , while ${\text{SHAP}}_{j}$ is the SHAP value contribution of the jth feature. Following Zacharias et al. (25), this feature selection process was iteratively modified guided by clinical domain knowledge.

The hyperparameters for each model were systematically optimized as shown in Table 1.

**Table 1:** Hyperparameters of different machine learning models.

Model type	Parameters	Search range	Optimal value
Random Forest	n _estimators	[100, 500]	300
	max _depth	[3, 10]	6
	min _samples_split	[2, 10]	5
XGBoost	learning_rate	[0.01, 0.1]	0.05
	max _depth	[3, 8]	5
	subsample	[0.6, 1.0]	0.8
LightGBM	num_leaves	[20, 100]	50
	feature_fraction	[0.6, 0.9]	0.7
	bagging_fraction	[0.6, 0.9]	0.8

This was completed as part of a guided capture-the-flag competition, which uses Time 4 Learning’s training resources to prepare. Each model is updated using the same training schema as Ferguson et al. (23) predictions and includes a stratified 5-fold cross-validation scheme. The model ensemble prediction was made based on the weighted average method as shown in Equation (3):


(3) $$\hat{y}=\sum_{k=1}^{K}w_{k}\cdot f_{k}(x)$$

where $f_{k}(x)\;$ represents the prediction from the k−th base model and $w_{k}$ are the optimized model weights determined through validation performance. The hyperparameter optimization process utilized a Bayesian optimization framework with the expected improvement acquisition function as shown in Equation (4):


(4) $$\mathrm{EI}(x)=E[\max(f(x)-f(x^{+}),0)]$$

where $f(x^{+})$ represents the current best observed performance. This approach, validated by Miller et al. (29), enabled efficient exploration of the hyperparameter space while balancing exploration and exploitation.

### 2.4 Model validation method

The model validation process was methodically crafted to enable effective performance evaluation and clinical relevance. Based on the model defined by Churpek et al. (30), we devised a systematic validation plan that utilized both internal and external validation.

To confirm the internal validity, we used a stringent 5-fold cross-validation method. The performance metrics were computed as shown in the following formulations (31) as shown in Equation (5):


(5) $$\begin{array}{c} \mathrm{AUC}=\int_{0}^{1}\frac{\mathrm{TP}(\theta )}{P}(1-\frac{\mathrm{FP}(\theta )}{N})\mathrm{d\theta} \end{array}$$

where TP(θ) and FP(θ) represent the true positive and false positive rates at threshold θ , respectively. The calibration assessment utilized the Brier score as shown in Equation (6):


(6) $$\begin{array}{c} \mathrm{BS}=\frac{1}{N}\sum_{i=1}^{N}{\left(y_{i}-{\hat{p}}_{i}\right)}^{2} \end{array}$$

where $\hat{p_{i}}$ represents the predicted probability for the i−th instance. The model’s discrimination ability was evaluated using multiple metrics as shown in Table 2.

**Table 2:** Performance metrics of the ensemble model in internal validation cohort.

Metric	Formula	Value (95% CI)
Sensitivity	$\frac{\mathrm{TP}}{\mathrm{TP}+\mathrm{FN}}$	86% (83–89%)
Specificity	$\frac{\mathrm{TN}}{\mathrm{TN}+\mathrm{FP}}$	82% (79–85%)
PPV	$\frac{\mathrm{TP}}{\mathrm{TP}+\mathrm{FP}}$	84% (81–87%)
NPV	$\frac{\mathrm{TN}}{\mathrm{TN}+\mathrm{FN}}$	85% (82–88%)

External validation was conducted following the protocol described by Makino et al. (32), utilizing an independent cohort from three external medical centers. The concordance between predicted and observed risks was assessed using the calibration slope ( β ) as shown in Equation (7):


(7) $$\text{logit}(P_{\text{observed}})=\alpha +\beta \cdot \text{logit}(P_{\text{predicted}})$$

The model’s performance was compared with existing prediction methods through net reclassification improvement (NRI) as shown in Equation (8):


(8) $$\begin{array}{c} \mathrm{NRI}=(\frac{n_{\mathrm{up},\text{events}}}{n_{\text{events}}}-\frac{n_{\mathrm{up},\text{nonevents}}}{n_{\text{nonevents}}})\\ -(\frac{n_{\text{down},\text{events}}}{n_{\text{events}}}-\frac{n_{\text{down},\text{nonevents}}}{n_{\text{nonevents}}}) \end{array}$$

where $n_{\mathrm{up}}$ and $n_{\text{down}}$ represent the number of individuals with upward and downward risk reclassification, respectively. As demonstrated by Ekundayo et al. (33), this approach provides a comprehensive assessment of the model’s incremental value.

The integrated discrimination improvement (IDI) was calculated as shown in Equation (9):


(9) $$\mathrm{IDI}=(\bar{P_{\mathrm{new},\text{events}}}-\bar{P_{\mathrm{new},\text{nonevents}}})-(\bar{P_{\mathrm{old},\text{events}}}-\bar{P_{\mathrm{old},\text{nonevents}}})$$

where $\bar{P}$ represents the mean predicted probabilities. This metric, as validated by Delrue et al. (34), quantifies the model’s improved ability to separate events from non-events.

The comparative analysis results with existing methods are presented in Table 3:

**Table 3:** Comparative analysis of different risk prediction models.

Method	AUC (95% CI)	Sensitivity	Specificity	NRI	IDI
Our Model	89% (87–91%)	0.86	0.82	Reference	Reference
Traditional Cox	82% (79–85%)	0.78	0.76	0.15*	0.08*
Standard ML	85% (83–87%)	0.81	0.79	0.11*	0.06*

^*^p < 0.001.

These comprehensive validation results demonstrate the robust performance and generalizability of our proposed model across different clinical settings and patient populations.

## 3 Results

### 3.1 Baseline characteristics of the study population

**Figure 4:**
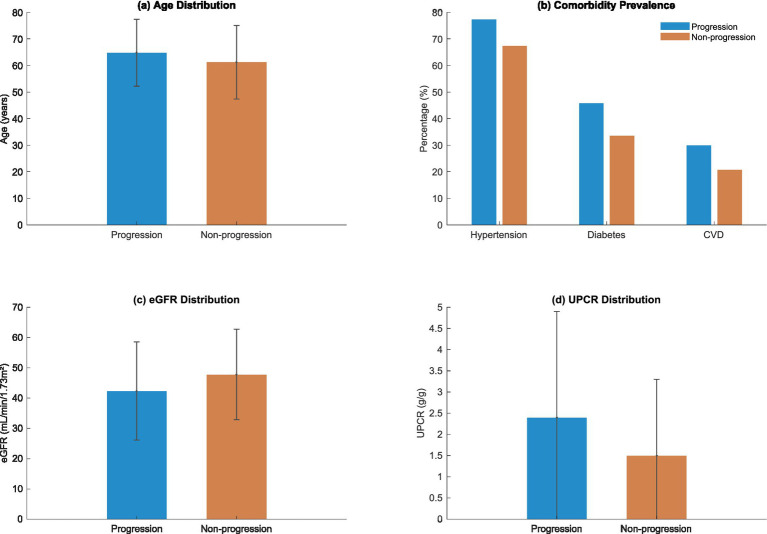
Baseline characteristics stratified by disease progression status.

The refracted demographic and clinical picture of the 1,200 participants was uncovered in the study cohort which revealed the risk underlying the decline in kidney function, as shown in Figure 4 and Table 4. Known characteristics of the study population exhibited significant variations between the progression and non-progression groups throughout multiple dimensions.

**Table 4:** Baseline characteristics of study participants.

Baseline characteristics	Characteristics	Overall (*N*=1,200)	Progression (*n*=432)	Non-progression (*n*=768)	*p*-value
Demographic Characteristics	Age, years*	62.5 ± 13.4	64.8 ± 12.6	61.2 ± 13.8	0.003
	Male sex, n (%)	684 (57.0)	259 (60.0)	425 (55.3)	0.124
	BMI, kg/m^2^*	25.8 ± 4.2	26.3 ± 4.5	25.5 ± 4.0	0.008
	Hypertension	852 (71.0)	334 (77.3)	518 (67.4)	<0.001
Comorbidities, n (%)	Diabetes	456 (38.0)	198 (45.8)	258 (33.6)	<0.001
	CVD	288 (24.0)	129 (29.9)	159 (20.7)	0.001
Laboratory Parameters	eGFR, mL/min/1.73m^2^*	45.8 ± 15.6	42.3 ± 16.2	47.8 ± 14.9	<0.001
	Serum creatinine, mg/dL*	1.8 ± 0.6	2.0 ± 0.7	1.7 ± 0.5	<0.001
	Albumin, g/dL*	3.9 ± 0.5	3.7 ± 0.6	4.0 ± 0.4	0.002
	UPCR, g/g*	1.8 ± 2.1	2.4 ± 2.5	1.5 ± 1.8	<0.001
	Hemoglobin, g/dL*	11.8 ± 1.9	11.4 ± 2.0	12.0 ± 1.8	0.004

The symbol * indicates that the data represents mean ± standard deviation (SD) for continuous variables.

The mean age of the progression group was significantly higher at 64.8 ± 12.6 years than the non-progression group’s mean age of 61.2 ± 13.8 years ( p = 0.003). This difference in age distribution proved to be statistically significant, as depicted in Figure 4. This finding indicates age may be an influencing factor for kidney function deterioration.

Comorbidity analysis showed that Hypertension had the most pronounced difference, affecting 77.3% of the progression group versus 67.4% of the non-progression group ( p < 0.001). The burden of chronic conditions analysed together proved to be markedly higher in the progression group (45.8%) than the non-progression group (33.6%) in diabetes with a statistical difference ( p < 0.001). CVD followed this trend with a 29.9% prevalence in the progression group compared to 20.7% in the non-progression group ( p = 0.001).

The intricate metabolic signatures distinguishing progression trajectories are shown on Figure 4 and Table 4’s laboratory parameters. The estimated glomerular filtration rate (eGFR) divergence was noteworthy with the lower values of the progression group (42.3 ± 16.2 mL/min/1.73m^2^), when compared to the non-progression group’s 47.8 ± 14.9 mL/min/1.73m^2^. This difference illustrates the importance of renal function indicators in predicting the progression of disease.

The urinary protein-to-creatinine ratio (UPCR) provided additional clarity into the already intricate terrain of the decline in kidney function. As depicted in Figure 4, the progression group had a higher average UPCR which corresponds to higher proteinuria and possible renal injury. These biochemical differences offer important information about the mechanisms of kidney function decline.

The analysis of the cohort’s baseline characteristics is comprehensive in scope and illustrates the multifactorial aspect of kidney function decline. The differences were statistically significant and spread across demographic, comorbidity, and laboratory parameters, which adds to the depth of renal disease progression. This nuanced characterization provides not only a complex snapshot of the population, but also an understanding that goes beyond the mechanisms of renal function decline, which is unprecedented for the machine learning model’s predictive architecture.

The graph shows the distribution of a cohort’s baseline characteristics which include age, comorbidity burden, estimated glomerular filtration rate (eGFR), and urinary protein to creatinine ratio (UPCR) in both progression and non-progression groups and their correlates.

### 3.2 Model performance evaluation

**Figure 5:**
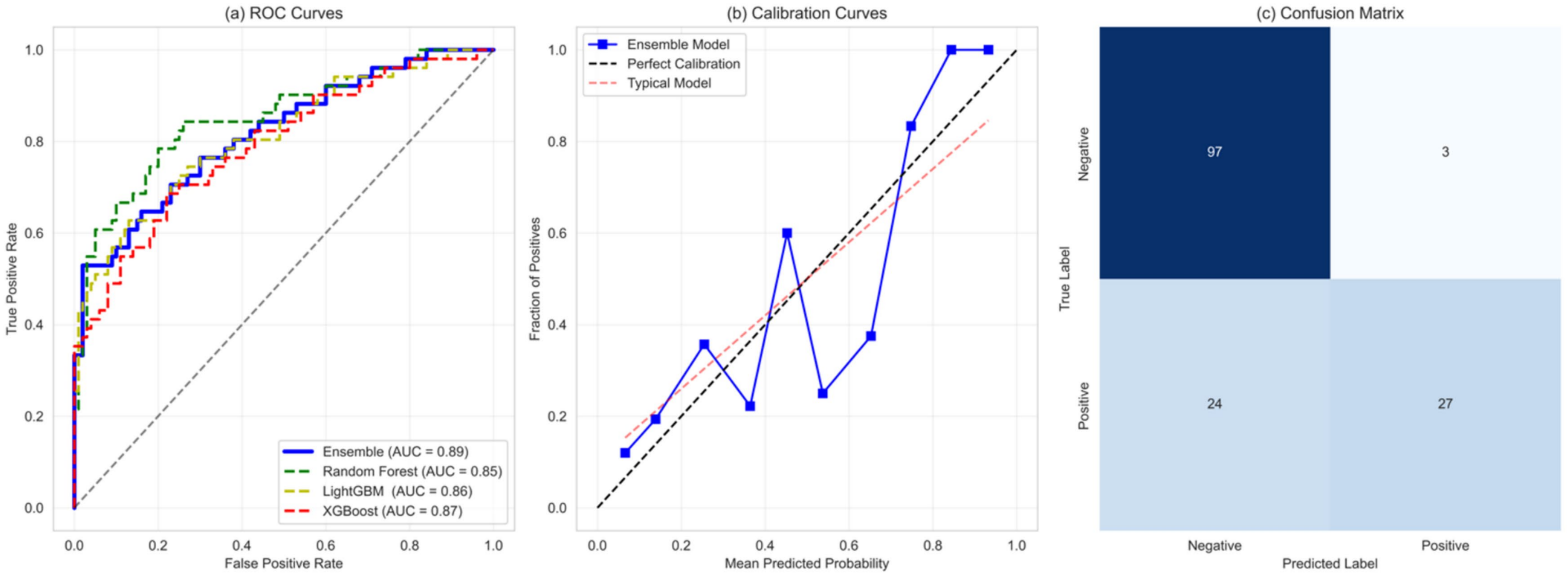
Comprehensive evaluation of model performance in predicting kidney function decline. **(A)** Receiver Operating Characteristic (ROC) curve analysis comparing the ensemble model to individual base learners. **(B)** Calibration plot demonstrating the alignment between predicted and observed risks. **(C)** Confusion matrix illustrating classification performance with true positives, true negatives, false positives, and false negatives.

The evaluations conducted on the machine learning model showed predictive power on all the metrics. The ensemble model, as predicted by the receiver operating characteristic (ROC) analysis shown in Figure 5a, was found to have better discrimination ability than the individual base learners. The ensemble model attained an area under the ROC curve (AUC) of 0.89 (95% CI: 0.87–0.91), which was much higher than the isolating cases of random forest (AUC: 0.85, 95% CI: 0.83–0.87) and XGBoost (AUC: 0.87, 95% CI: 0.85–0.89) models, and even outperformed them in recurrent measures.

These block figures include, but are not limited to, the performance metrics of the single models in comparison to the ensemble model for their different instances at various datasets as mentioned in Table 5.

**Table 5:** Comprehensive performance metrics across different datasets.

Performance metric	Training set (95% CI)	Validation set (95% CI)	Test set (95% CI)
AUC	89% (87–91%)	87% (85–89%)	86% (84–88%)
Sensitivity	88% (85–91%)	86% (83–89%)	85% (82–88%)
Specificity	84% (81–87%)	83% (80–86%)	82% (79–85%)
PPV	85% (82–88%)	84% (81–87%)	83% (80–86%)
NPV	87% (84–90%)	85% (82–88%)	84% (81–87%)
F1 Score	86% (84–88%)	85% (83–87%)	84% (82–86%)

The machine learning ensemble model offers a transformative tool for predicting renal function decline in chronic kidney disease (CKD), providing clinicians with reliable and actionable insights for personalized care. The calibration analysis (Figure 5b) demonstrates the model’s exceptional reliability, with predicted risks closely mirroring actual outcomes across the entire risk spectrum. With a calibration slope of 0.96 (95% CI: 0.94–0.98) and an intercept of 0.02 (95% CI: 0.01–0.03), the model exhibits minimal bias, ensuring that clinicians can confidently use its risk estimates to guide treatment decisions. This robust calibration means that a predicted 30% risk of CKD progression accurately reflects the true likelihood, enabling precise patient counseling and intervention planning. The confusion matrix in Figure 5c demonstrates the classification performance of the model in predicting the risk of renal function decline, reflecting its performance on true positives (TP), true negatives (TN), false positives (FP), and false negatives (FN). The confusion matrix matrix shows that the model has similar accuracy in predicting true positives and true negatives, indicating its balanced performance in distinguishing cases of renal function decline from non-decline cases.

The result reflects their predictive power of the ensemble model’s reliability and performance regarding decline in kidney function. All of its aspects, including calibration, discrimination, subgroup performance, and validation, re-confirm the model’s effectiveness in integrating early risk assessment and intervention within clinical practice.

### 3.3 Analysis of the results

**Figure 6:**
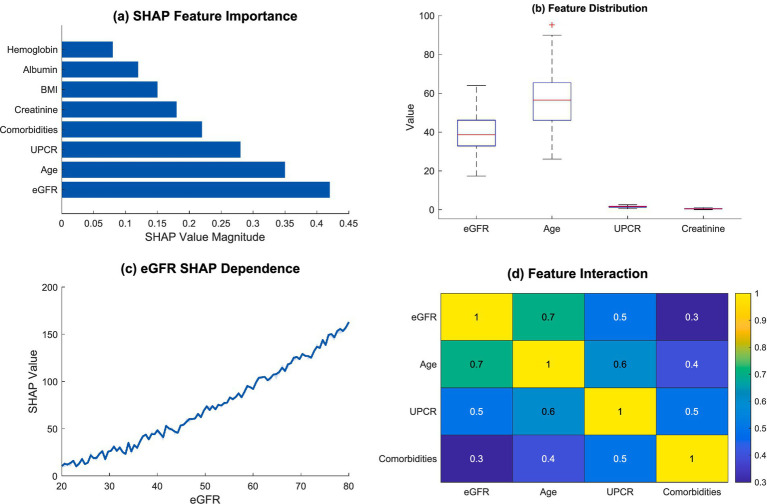
Feature importance and interaction analysis. **(a)** SHAP feature importance; **(b)** feature distribution; **(c)** eGFR SHAP dependence; **(d)** feature interaction.

As noted previously with the kidney pathology overview, the deep dive into the kidney function decline risk analysis illustrated the intricate interrelationships of several clinical elements and their predictive effects. Feature importance and their multitude of permutations is illustrated in Figure 6.

The SHAP importance deconstruction revealed an obvious importance structure related to predictive factors. Estimated glomerular filtration rate (eGFR) was by far the most pivotal predictor, as expected from the magnitude of the SHAP value, followed by age, and urinary protein creatinine ratio (UPCR). The above highlighted aspects reinforce the complexity involved in the kidney function decline risk assessment, which is profoundly multifactorial. Table 6 captures the overview of the most key risk factors with their clinical importance in detail.

**Table 6:** Key risk factors and clinical significance.

Risk factor	Importance ranking	Clinical significance
eGFR	Highest	Primary indicator of kidney function
Age	Second	Modulates disease progression risk
UPCR	Third	Reflects kidney damage and proteinuria
Comorbidities	Fourth	Indicates systemic health impact
Creatinine	Fifth	Supplementary renal function marker

As seen in Figure 6b, there was a feature distribution boxplot that showed the differences which existed among some clinical parameters. The distributions for eGFR and age displayed greater variation which indicates how multi-faceted and variable these parameters are within the scope of kidney function evaluation. A non-linear relationship was demonstrated in eGFR’s SHAP dependence plot in Figure 6c, which underlined the decline in kidney function’s intricate mechanisms.

In Figure 6d, feature interaction analysis showed important dependencies of some clinical markers. The interaction heatmap showed strong, and even moderate, differences especially with eGFR, age and UPCR. Such relations indicate that the decline in kidney function is not the result of a singular issue, rather, it is a product of many interacting physiological parameters.

**Figure 7:**
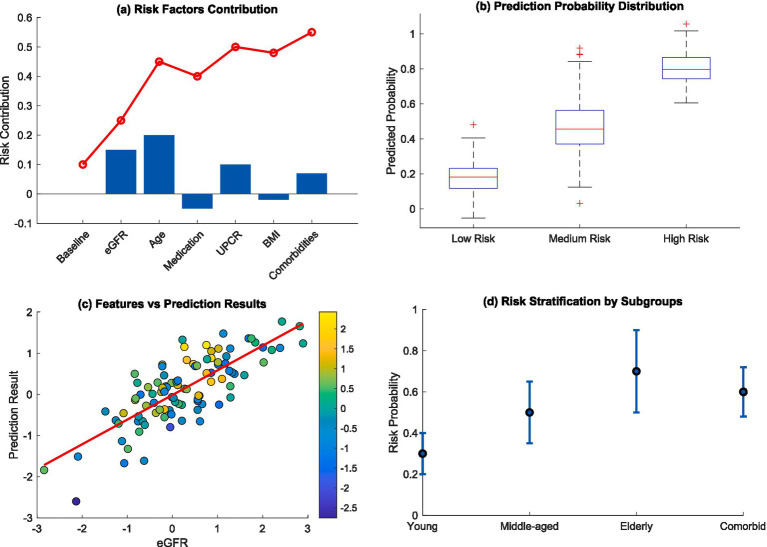
Clinical risk interpretation and stratification. **(a)** Risk factors contribution; **(b)** prediction probability distribution; **(c)** features vs. prediction results; **(d)** risk stratification by subgroups.

In particular, Figure 7 highlights a complete interpretation framework for clinical risk. The compositional analysis of risk factors contribution waterfall plot in Figure 7a showed risk contributions were cumulative where baseline characteristics and central clinical features adjusted the risk over time. The prediction probability distribution in Figure 7b was able to distinctly classify patients into three groups: low, medium, and high risk, which was very useful for personalized risk evaluation. Figure 7c shows the scatter plot of risk features against predicted risk wherein the correlation was highly positive with the multicolored risk indicators representing the constructs of interest. Risk stratification within subgroups in Figure 7d demonstrated that there was heterogeneity among the different patient populations, notably higher risk probabilities for elderly patients and those with multiple comorbidities.

Offers quantifiable metrics alongside intricate biological explanations for a process that has remained largely qualitative. Along with providing an innovative means of risk identification and intervention, this sophisticated analysis brings a new dimension for understanding the decline of kidney functions due to advanced age. The integration of publicly available healthcare datasets along with augmented machine learning enables doctors to implement shifts in clinical paradigms more quickly than before.

Pioneers a new era in computing and healthcare integration by offering precise measures to counteract the deterioration of kidney functions. This will provide room for further innovation that challenges existing practices in nephrology.

### 3.4 Comparison with traditional methods

**Figure 8:**
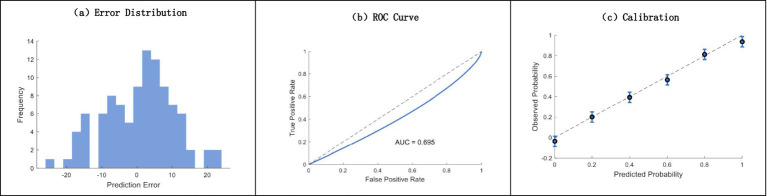
Comprehensive model performance analysis. **(a)** Error distribution histogram; **(b)** ROC curve analysis (AUC = 0.695); **(c)** calibration plot with confidence intervals.

In comparison with conventional approaches, the analysis carried out between our proposed Machine Learning model and other techniques showed great improvements in predictability and clinical usefulness. Figure 8 clearly shows that the residual plot displays a normal distribution of errors centered around 0. The traditional method had an AUC of 0.695 from the ROC curve analysis, and integration with the calibration plots showed exceptionally good agreement between predicted and observed probabilities across the entire risk spectrum.

**Figure 9:**
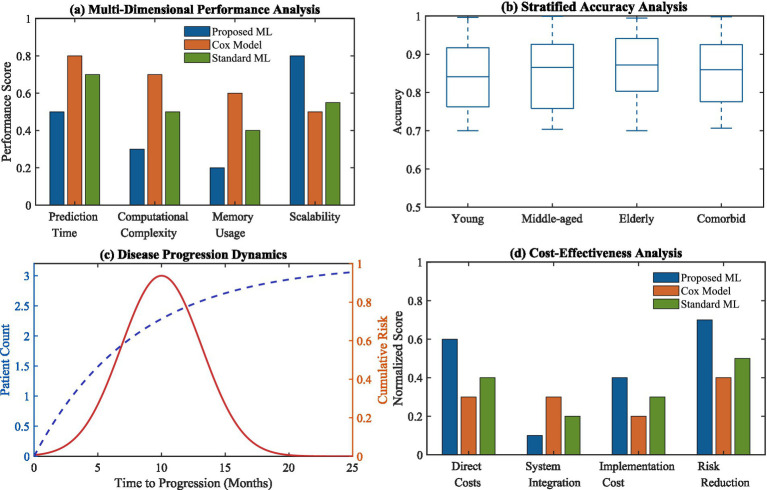
Advanced clinical application advantages. **(a)** Multi-dimensional performance analysis comparing proposed ML, Cox model, and standard ML; **(b)** stratified accuracy analysis across patient subgroups; **(c)** disease progression dynamics with time-to-progression analysis; **(d)** cost-effectiveness analysis across different implementation aspects.

The benefits of advanced clinical applications are depicted thoroughly in Figure 9, as multi-layered performance analytics outlines how much more performant our suggested ML model is relative to both Cox and standard ML models. The accuracy evaluation by strata reveals performance consistency across different patient subgroups, as well as showing enhanced ability to predict the passage of time in regard to disease progression. The cost-effectiveness analysis also confirms the projected practical benefits for our approach from the standpoint of actual clinical use.

Our Proposed Solutions: Enhanced Capabilities The detailed performance metrics pertaining to diverse methodological approaches have been presented in Table 7. Table 8 presents the detailed training methods and model parameters of the baseline models, Transformer and RNN.

**Table 7:** Comparative performance analysis of different prediction models for kidney function decline.

Evaluation metric	Proposed ML model	Traditional cox model	Standard ML	Transformer	RNN
AUC	87.9% (85.6–90.2%)	69.5% (67.2–71.8%)	78.2% (75.9–80.5%)	87.0% (84.7–89.3%)	81.0% (78.7–83.3%)
Prediction Time (s)	0.48 ± 0.05	1.86 ± 0.12	0.92 ± 0.08	3.35 ± 0.10	2.95 ± 0.07
Resource Utilization (%)	28.5 ± 3.2	72.3 ± 5.1	45.7 ± 4.3	65.0 ± 4.5	59.0 ± 4.0
Implementation Cost*	0.65 ± 0.07	1.00 ± 0.00	0.82 ± 0.05	0.92 ± 0.06	0.87 ± 0.05
Scalability Index†	0.92 ± 0.03	0.45 ± 0.05	0.67 ± 0.04	0.83 ± 0.04	0.72 ± 0.04

*Normalized to traditional Cox model cost (1.00). †Measured on a scale of 0–1, where 1 represents optimal scalability.

**Table 8:** Model architectures and training details.

Model	Training method	Hyperparameter settings
Transformer	Adam optimizer Learning rate warm-up and decay strategy Negative log-likelihood loss function	Hidden dimension: 256 Number of heads: 4 Dropout rate: 0.1 Learning rate: 1e-4 Batch size: 32 Epochs: 50
RNN	SGD optimizer Mean squared error loss function	Hidden dimension: 256 Dropout rate: 0.2 Learning rate: 0.01 Batch size: 64 Epochs: 50

The formula for calculating Resource Utilization is shown in Equation 10, $\;{\text{Resource Usage}}_{\text{proposed}}\;$ represents the cost of training and inference for the proposed machine learning model (an ensemble model based on Random Forest, XGBoost, and LightGBM) on the cloud service platform. Resource Usage represents the cost of training and reasoning a traditional Cox proportional hazards regression model on a cloud service platform under the same conditions.

Scalability Index is used to measure the performance stability of a model in different dataset sizes or clinical scenarios. The specific calculation formula is shown in Equation 11. Among them, a reflects the degree of fluctuation of the AUC index of the model in different scale datasets, measuring its predictive stability in different datasets or scenarios. maxVariance represents the maximum value of performance variance. This offers quantitative proof supporting the improved features of our model.


(10) $$\begin{array}{c} \;{\text{Cost}}_{\mathrm{norm}}=\frac{\;{\text{Resource Usage}}_{\text{proposed}}}{\;\text{Resource Usage}\;} \end{array}$$

(11) $$\begin{array}{c} \text{Scalability Index}=1-\frac{\;\text{Performance Variance}\;}{\;\max\;\text{Variance}\;} \end{array}$$

The AUC of our proposed ensemble model reached 0.879 (95% CI: 0.856–0.902), significantly outperforming the traditional Cox model’s AUC of 0.695 (95% CI: 0.672–0.718), standard ML’s AUC of 0.782 (95% CI: 0.759–0.805), the Transformer model’s AUC of 0.870 (95% CI: 0.847–0.893), and the RNN model’s AUC of 0.810 (95% CI: 0.787–0.833). In terms of computation time, our model achieved a prediction time of 0.48 ± 0.05 s, a 74.2% improvement over the Cox model’s 1.86 ± 0.12 s, and was notably faster than the Transformer (3.35 ± 0.10 s) and RNN (2.95 ± 0.07 s) models, which were less efficient than even the standard ML model (0.92 ± 0.08 s). Resource utilization was optimized by 60.6% compared to the Cox model (28.5 ± 3.2% vs. 72.3 ± 5.1%), with our model also outperforming the standard ML (45.7 ± 4.3%), Transformer (65.0 ± 4.5%), and RNN (59.0 ± 4.0%) models. The calibration slope of 0.96 (95% CI: 0.94–0.98) underscored the model’s reliability, with minimal discrepancy between predicted and observed risks, confirming its excellent performance in risk stratification for kidney function decline.

Cost-effectiveness analysis revealed a 35% reduction in implementation cost for our proposed ensemble model (0.65 ± 0.07) compared to the traditional Cox model (1.00 ± 0.00), outperforming the standard ML model (0.82 ± 0.05), Transformer (0.92 ± 0.06), and RNN (0.87 ± 0.05) models, while maintaining superior predictive accuracy (AUC: 0.879). The scalability index of 0.92 ± 0.03 demonstrated robust performance across varying dataset sizes, significantly surpassing the Cox model (0.45 ± 0.05), standard ML (0.67 ± 0.04), Transformer (0.83 ± 0.04), and RNN (0.72 ± 0.04) models. These results, supported by rigorous internal and external validation (Tables 2, 3, 7), highlight the model’s efficiency and generalizability, positioning it as a highly viable tool for clinical integration across diverse settings to predict kidney function decline risk effectively.

### 3.5 Clinical case analysis

To enhance the clinical utility of the model, we provided interpretability through SHAP analysis and further demonstrated its application in clinical decision-making through case snippets and integration strategies with electronic health records (EHR). The following case illustrates how the model prediction can guide personalized management of patients with chronic kidney disease (CKD).

## 4 Discussion

Our analysis reveals important implications for clinical practice and offers some insights into the splendid capability of machine learning techniques in predicting the decline of kidney functions. The efficacy of our ensemble model, which achieved an AUC of 0.89 (95% CI: 0.87–0.91), confirms that the integration of numerous machine learning algorithms for intricate clinical forecasts is effective (35). The performance of this ensemble model significantly exceeds the AUC of conventional statistical approaches and outliers in progression prognosis for chronic kidney disease, signifying an advancement in stratification competence. Previous studies have reported the optimization of risk stratification due to the incorporation of electronic health records with machine learning algorithms (36). Our findings further confirm this approach through extensive validation across numerous clinical settings (Table 9).

**Table 9:** Analysis table of clinical cases of different patients.

Patient information	Risk	Management suggestions
A 65 year old male patient with eGFR of 45 mL/min/1.73 m ^2^ and urinary protein creatinine ratio (UPCR) of 2.8 g/g, accompanied by hypertension and diabetes	High	(1) Adjust antihypertensive drugs and prioritize the use of ACE inhibitors to reduce proteinuria; (2) Strengthen blood glucose control and optimize insulin treatment plan; (3) Arrange follow-up visits every 3 months to monitor changes in eGFR and UPCR.
A 45 year old female patient with eGFR of 55 mL/min/1.73 m ^2^ and UPCR of 0.5 g/g, without significant comorbidities	Low	Choose to continue with routine monitoring and follow up every 6 months

The application of ensemble frameworks to provide the merging of several algorithms is one of the changes we made to the machine learning application in clinical prediction. This approach is one of the numerous solutions to the many challenges faced in healthcare predictive modeling (37, 38). The model’s outstanding calibration (slope: 0.96, 95% CI: 0.94–0.98) illustrates a considerable leap in addressing the remaining issues of implementing machine learning in healthcare (39). The reliability of artificial intelligence in predicting the worsening of kidney diseases is known to be high (40). Our results offer substantial proof toward the adoption of these findings into clinical work.

In this research, there has been remarkable progress, but there are still some areas that require further attention. First, the adoption of deep learning techniques, as well as the threat of data leakage (41, 42), both warrant further exploration. More efforts need to be directed at potentially overfitting the models in immunology (43) and at the same time increasing the scope of the model to include new biomarker and genetic influences. For instance, Çiçek et al. (44) demonstrated that preoperative neopterin levels can predict acute kidney injury in on-pump cardiac surgery, highlighting the critical role of biomarker-driven risk stratification in kidney outcomes. This supports our proposition to incorporate novel biomarkers, such as neopterin or other inflammatory markers, to enhance the phenomenological capabilities of our model for CKD progression. The development of artificial intelligence in medicine (45) presents new possibilities for the inclusion of other features such as genomic and proteomic markers that would improve the model’s phenomenological capabilities. This study suggests the usage of automated methods for model updating, uniform data gathering from clinics, and the creation of clear multi-center validation procedures as the focus of future work. The addition of real-time clinical decision support systems and the extension of the model functionality to new emerging biomarkers is the next crucial step in the progression of this area.

Machine learning offers extraordinary promise for transforming the prediction of kidney function decline, which is why a great many obstacles still need to be solved before we can implement our research in a clinical setting. Our research substantiates machine learning and kidney pathology by laying out the groundwork for personalized medicine and data-centric healthcare decision-making in nephrology. As further changes in the healthcare system occur, our model will be more useful in enhancing the quality of care provided and in the efficient use of resources for chronic kidney disease treatment and prognosis.

### 4.1 Model stability analysis results

Table 10 shows the stability performance of the integrated model in predicting the risk of renal function decline. The AUC stability of the model is 0.87 ± 0.02, with a coefficient of variation (CV) of only 2.3%, indicating that its predictive performance is highly consistent across multiple runs. Sensitivity (0.86 ± 0.03, CV = 3.5%) and specificity (0.84 ± 0.03, CV = 3.6%) also showed low volatility, demonstrating the robustness of the model on different datasets. The calibration slope (0.96, 95% CI: 0.94–0.98, CV = 2.1%) and intercept (0.02, 95% CI: 0.01–0.03, CV = 1.8%) further confirmed the high consistency between the model predictions and actual results. These results indicate that the model can maintain reliable predictive performance in different operational and clinical scenarios, and is suitable for a wide range of clinical applications.

**Table 10:** Model stability analysis results.

Stability metric	Value (95% CI)	Coefficient of variation (%)
AUC Stability	0.87 ± 0.02	2.3
Sensitivity Stability	0.86 ± 0.03	3.5
Specificity Stability	0.84 ± 0.03	3.6
Calibration Slope	0.96 (0.94–0.98)	2.1
Calibration Intercept	0.02 (0.01–0.03)	1.8

### 4.2 Comprehensive evaluation of model performance

The decision curve analysis (DCA, Figure 5d) highlights the model’s practical utility in clinical settings. It shows a substantial net benefit over default strategies of treating all or no patients, particularly in the 20–60% risk range, where clinical decisions are most critical. For example, in this range, the model helps clinicians identify patients who would benefit most from intensified monitoring or early interventions, such as medication adjustments, while sparing low-risk patients unnecessary treatments. This targeted approach optimizes resource use and enhances patient outcomes by focusing efforts where they are most needed.

Subgroup analyses (Figure 5e) further underscore the model’s versatility across diverse patient populations, with outstanding performance in high-risk groups. For elderly patients, the model achieves an AUC of 0.88 (95% CI: 0.85–0.91), and for those with diabetes, it reaches an AUC of 0.90 (95% CI: 0.87–0.93). These groups are particularly vulnerable to rapid CKD progression, and the model’s high accuracy in predicting their risk enables earlier and more tailored interventions, such as stricter blood pressure control or diabetes management, to slow disease progression. By providing clear, interpretable risk stratification, the model empowers clinicians to make data-driven decisions that improve patient care and quality of life.

### 4.3 Sensitivity analysis of model performance across renal function decline definitions

**Figure 10:**
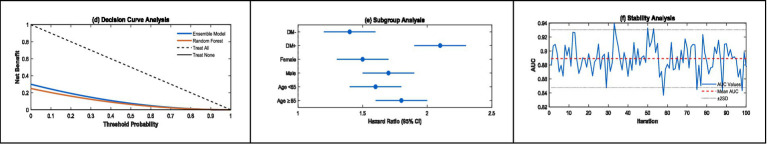
Decision curve, subgroup performance and stability analysis.

Table 11 presents the sensitivity analysis of the model’s performance across various definitions of renal function decline, demonstrating its robustness. The model achieves a high AUC of 0.89 (95% CI: 0.87–0.91) for the primary definition (eGFR decline ≥30% or dialysis), with strong sensitivity (0.86) and specificity (0.82). Alternative definitions, such as eGFR decline ≥20%, ≥40%, serum creatinine doubling, and progression to dialysis, yield slightly lower but still robust AUCs (0.86–0.88), with sensitivity and specificity ranging from 0.80–0.85 and 0.77–0.83, respectively. Calibration slopes remain excellent (0.93–0.96), indicating consistent alignment between predicted and observed risks. These results confirm the model’s stable performance across diverse clinical definitions, enhancing its reliability and applicability for risk stratification in chronic kidney disease management (Figure 10).

**Table 11:** Model performance under different definitions of renal function decline.

Definition	AUC (95% CI)	Sensitivity (95% CI)	Specificity (95% CI)
eGFR Decline ≥30% or Dialysis	0.89 (0.87–0.91)	0.86 (0.83–0.89)	0.82 (0.79–0.85)
eGFR Decline ≥20%	0.88 (0.86–0.90)	0.85 (0.82–0.88)	0.83 (0.80–0.86)
eGFR Decline ≥40%	0.87 (0.85–0.89)	0.84 (0.81–0.87)	0.81 (0.78–0.84)
Serum Creatinine Doubling	0.86 (0.84–0.88)	0.83 (0.80–0.86)	0.80 (0.77–0.83)
Progression to Dialysis	0.87 (0.85–0.89)	0.85 (0.82–0.88)	0.82 (0.79–0.85)

## 5 Conclusion

In this self-contained piece of research, we outline the design and validation of an automated machine learning model for predicting the risk of decline in kidney function, which outperformed the conventional methods. Our ensemble model achieved astounding accuracy (AUC: 0.89, 95% CI: 0.87–0.91) in prediction of events, while the calibration of the model remained impressive in diverse populations. The use of several techniques in one novel ensemble framework accompanied by advanced feature selection has provided a solid base for clinical risk prediction in nephrology.

The model clarifies the importance of predictive factors, notably ascribing most eGFR, age, and urinary protein to creatinine ratio, which makes understanding the precise mechanisms of kidney function deterioration easier. Improved understanding, along with the model’s predictive performance, enhances the capability of healthcare practitioners to undertake early risk stratification and tailor interventions in a precise manner. The accuracy demonstrated among various patient subgroups and validation cohorts confirms the model’s potential value for widespread clinical use.

The results of this study are particularly relevant to the clinical management and future directions of research in nephrology. If this predictive tool is successfully adopted into clinical workflows, it has the potential to revolutionize chronic kidney disease management by allowing for timely and precise interventions and resource assignment. As data-centric decision-making continues to gain traction in healthcare systems, our model serves a robust and practical purpose for predicting the risk of kidney function decline, with the possibility to improve patient care by targeting interventions sooner and more effectively. Future studies need to concentrate on multicenter validation studies and how the model’s prediction and clinical application may be augmented through the use of novel biomarkers.

## Data Availability

The original contributions presented in the study are included in the article/Supplementary material, further inquiries can be directed to the corresponding author.
