# Supplementary material for: Ensemble machine learning for predicting renal function decline in chronic kidney disease: development and external validation
Source: Front Med (Lausanne). 2025 Oct 27;12:1598065. doi: 10.3389/fmed.2025.1598065 (PMC12598044; doi:10.3389/fmed.2025.1598065)
Supplement: Supplementary file 1 [file Table_1.docx]

**Appendix**

**Table 12: TRIPOD List**

| **Section** | Item | | Checklist item |
| --- | --- | --- | --- |
| **TITLE** |  |  |  |
| Title | 1 | D/V | Identify the study as developing or evaluating the performance of a multivariable prediction model, the target population, and the outcome to be predicted. |
| **ABSTRACT** |  |  |  |
| Abstract | 2 | D/V | Covering key information such as research background, purpose, methods, results, and conclusions |
| **INTRODUCTION** |  |  |  |
| Research Background | 3a | D/V | Elaborate on the background of CKD and the necessity of developing new predictive models |
| Research Objectives | 3b | D/V | Specify the objectives, including whether the study describes the development, validation, or updating of the model. |
| **METHODS** |  |  |  |
| Data Source | 4a | D/V | Describe the study design or source of data (e.g., cohort, randomized trial, or case-control), and the time period during which participants were recruited. |
| Time Range | 4b | D/V | Specify the key study dates, including start and end dates of recruitment. |
| Research Type Description | 4c | D/V | State whether the report describes a multivariable prediction model development and/or validation. |
| Data Preprocessing | 4d | D/V | Describe the data preprocessing and quality inspection process |
| Predicted Outcome | 4e | D/V | Specify the outcome(s) to be predicted, how it/they is/are defined, and its/their relevance to the target population. |
| Processing Method | 4f | D/V | List the predictive factors and their measurement methods |
| Handling of Predictive Factors | 4g | D/V | Describe the handling of predictive factors, including the handling of missing data |
| Model Construction | 4k | D/V | Describe the selection of predictive factors and the process of model construction |
| Participant Flow | 4e | D/V | Describe the flow of participants through the study. |
| Blinding in Outcome Assessment | 4j | V | Describe the blinding method used in the evaluation of the outcomes to prevent bias. |
| Model Performance | 4k | D/V | Report the performance indicators of the model |
| Pre Specified Predictive Factors | 4l | D/V | Pre specified predictive factors: Key predictive factors are listed to confirm consistency with clinical knowledge. |
| **RESULTS** |  |  |  |
| Basic Characteristics | 5a | D/V | Basic characteristics of report participants |
| Final Model Predictive Factors | 5b | D/V | List the predictive factors of the final model |
| Model Performance Metrics | 5c | D/V | Report on model performance metrics in development data |
| Report | 5d | V | Report validation data on model performance metrics |
| DISCUSSION |  |  |  |
| Discuss | 6f | D/V | Discuss the limitations of current research |

**Table 13: Baseline Characteristics Of External Queues**

| **Characteristic** | **Overall (n=600)** | **Progression (n=216)** | **Non-Progression (n=384)** |
| --- | --- | --- | --- |
| **Demographic Characteristics** |  |  |  |
| Age, years* | 63.2 ± 13.3 | 65.2 ± 12.8 | 61.8 ± 13.5 |
| Male sex, n (%) | 342 (57.0) | 130 (60.2) | 212 (55.2) |
| BMI, kg/m²* | 26.0 ± 4.3 | 26.5 ± 4.6 | 25.7 ± 4.1 |
| **Comorbidities, n (%)** |  |  |  |
| Hypertension | 440 (73.3) | 169 (78.2) | 271 (70.6) |
| Diabetes | 241 (40.2) | 100 (46.3) | 141 (36.7) |
| Cardiovascular Disease | 151 (25.2) | 66 (30.6) | 85 (22.1) |
| **Laboratory Parameters** |  |  |  |
| eGFR, mL/min/1.73m²* | 45.6 ± 15.8 | 41.8 ± 16.5 | 48.2 ± 15.1 |
| Serum Creatinine, mg/dL* | 1.9 ± 0.6 | 2.1 ± 0.7 | 1.7 ± 0.5 |
| Albumin, g/dL* | 3.9 ± 0.5 | 3.7 ± 0.6 | 4.0 ± 0.4 |
| Urinary Protein-to-Creatinine Ratio, g/g* | 1.9 ± 2.2 | 2.5 ± 2.6 | 1.6 ± 1.9 |
| Hemoglobin, g/dL* | 11.9 ± 1.9 | 11.5 ± 2.0 | 12.1 ± 1.8 |
